# Effect of menopause on the modified Rodnan skin score in systemic sclerosis

**DOI:** 10.1186/ar4587

**Published:** 2014-06-23

**Authors:** Évelyne Vinet, Sasha Bernatsky, Marie Hudson, Christian A Pineau, Murray Baron

**Affiliations:** 1 Division of Clinical Epidemiology, McGill University Health Centre, Montreal, Canada; 2 Division of Rheumatology, McGill University Health Centre, Montreal, Canada; 3 Division of Rheumatology, Jewish General Hospital, Montreal, Canada; 4 Lady Davis Institute for Medical Research, Montreal, Canada

## Abstract

**Introduction:**

We aimed to evaluate the effect of menopause on skin thickening, as measured by the modified Rodnan skin score (mRSS), in women with systemic sclerosis (SSc).

**Methods:**

We identified women with either limited or diffuse SSc, aged ≥ 18 years, enrolled within the Canadian Scleroderma Research Group (CSRG) cohort, between 2004 and 2011. As part of the CSRG cohort, subjects undergo annual assessments with standardized questionnaires and physical examinations. We performed multivariate regression analyses using generalized estimating equation (GEE) to determine the effect of menopause on the mRSS, adjusting for relevant covariates including notably age, follow-up time, and disease duration.

**Results:**

We identified 1070 women with SSc, contributing a total of 3546 observations over the study period. Of these women, at baseline, 65% had limited disease and 35% diffuse disease. In multivariate analyses, we observed a substantial effect of postmenopausal status on the mean mRSS in women with diffuse disease subtype [−2.62 units, 95% confidence interval (CI) -4.44, −0.80] and significant interaction between menopausal status and disease subtype (2.04 units, 95% CI 0.20, 3.88). The effect of postmenopausal status on the mean mRSS was smaller in women with limited SSc (−0.58, 95% CI −1.50, 0.34).

**Conclusions:**

Our results suggest that menopause has a substantial effect on skin thickening in diffuse SSc, with postmenopausal status being associated with a lower mean mRSS compared to premenopausal status.

## Introduction

Systemic sclerosis (SSc) is a multisystemic autoimmune disease, which predominantly affects women (with a female-to-male ratio of up to 9:1) [1]. Skin thickening is a defining feature of SSc, which is characterized by excessive production of extracellular matrix proteins (for example, collagen, laminin, fibronectin) by fibroblasts, resulting in skin thickening and internal organ fibrosis [1,2]. Currently, no therapy with an acceptable toxicity profile has proven to be effective in the treatment of SSc skin disease [3]. Studies have shown a potential effect of methotrexate, but they were limited by their small sample size [4,5]. Cyclophosphamide, a potent cytotoxic drug, was observed to have an effect on skin thickening when used for the treatment of severe interstitial lung disease [6]. However, cyclophosphamide can lead to significant adverse effects, including serious infections, premature gonadal failure (that is, early menopause), and long-term increased risk of malignancy [7,8].

Recent experimental evidence suggests that estrogen increases synthesis of extracellular matrix proteins in cultures of SSc skin fibroblasts [9]. Indeed, investigators have shown that estrogen induced a significant increase of fibronectin, collagen type I, and laminin synthesis in SSc fibroblasts compared to untreated fibroblasts. Moreover, an estrogen-receptor inhibitor (that is, tamoxifen) induced a significant decrease of these extracellular matrix proteins in cultures of SSc skin fibroblasts [9].

Menopause, characterized by a low estrogenic state, is associated with skin thinning, due to decreased extracellular matrix protein deposition by fibroblasts [10]. Thinning of the dermis often accompanies aging. However, most studies suggest that collagen loss is more closely related to postmenopausal status than chronologic age, reflecting hormonal changes [10]. Investigators have reported a mean decline in dermal collagen of approximately 1 to 2% per year after menopause [11]. Estrogen supplementation in postmenopausal women has been shown to increase skin thickness by increasing skin collagen content [12].

Although SSc most commonly occurs near the end of the reproductive period and predominantly affects women, no one has investigated the impact of menopause on skin involvement in women with SSc. Thus, we aimed to evaluate the effect of menopause on skin thickening, as measured by the modified Rodnan skin score (mRSS), in women with SSc.

## Methods

### Subjects

We used previously collected data from women with either limited or diffuse SSc, aged ≥18 years, enrolled within the Canadian Scleroderma Research Group (CSRG) cohort, between 2004 and 2011. The CSRG cohort is a national prospective cohort recruiting subjects with SSc at 15 sites across Canada, since 2004. SSc diagnosis in all subjects is clinically confirmed by a rheumatologist. As part of the CSRG cohort, subjects undergo annual assessments with standardized questionnaires (both patient self-reported and physician-reported) and a physical examination.

### Covariates and exposure of interest

We obtained information on baseline characteristics including race/ethnicity (defined as white, black, or other), age at disease onset, and disease duration (since first non-Raynaud disease manifestation), as well as time-varying covariates recorded at each annual visit, including menopausal status, disease subtype (limited versus diffuse), smoking (ever versus never smoker), and medications. Medication exposures were collected by physicians and included information on exposure (current versus past/never) to oral contraceptive pills (OCP), hormone replacement therapy (HRT), disease-modifying antirheumatic drugs (DMARDs) (that is, d-penicillamine, methotrexate, leflunomide, and mycophenolate mofetil, as well as bosentan), and exposure (ever versus never) to cyclophosphamide. Of note, bosentan was categorized a priori as a DMARD because observational studies have shown a potential beneficial effect on skin thickening [13,14], and cyclophosphamide was evaluated independently because of its well-known association with premature gonadal failure (that is, early menopause). The exposure of interest, menopausal status, was assessed through self-report, by asking the subject if they had reached menopause at baseline or since the last follow-up visit if they were previously premenopausal.

### Outcome assessment

The outcome was measured at each annual visit using the mRSS, a validated measure of skin thickening in SSc [15]. In the mRSS, skin thickening is assessed at 17 body sites by palpation and rated on a scale with values of 0 (normal), 1 (mild), 2 (moderate) or 3 (severe skin thickening) [2]. The total skin score is the sum of the individual skin assessments in the 17 body areas, with a possible range of 0 to 51; the higher the score, the greater the extent and severity of skin thickening.

### Statistical analysis

We used descriptive statistics to evaluate the subjects’ characteristics and the association between the different covariates. We performed univariate and multivariate regression analyses using a generalized estimating equation (GEE), with each subject serving as a cluster, and assuming an autoregressive correlation structure on the error terms, to determine the effect of menopause on the mRSS. The GEE method is an innovative statistical approach, which judiciously uses longitudinal data by directly relating changes in the covariate of interest (for example, menopausal status) in a given subject to changes in the outcome variable (for example, mRSS) of that same subject over time. Of note, missing data (present in fewer than 11% of visits) were handled by using multiple imputation.

In the multivariate analyses, in addition to menopausal status, other relevant covariates (defined above) were evaluated, and included: disease subtype, an interaction term between menopausal status and disease subtype, time (since baseline visit), disease duration (at baseline), age at disease onset, race/ethnicity, smoking, and medication exposures (that is, OCP, HRT, DMARDs, and cyclophosphamide). Furthermore, to investigate the effect of menopausal status on skin thickening in early disease, we conducted a subsample analysis, using the same multivariate model as specified above, but restricted to women with baseline disease duration shorter than 5 years.

The McGill University research ethics board approved this study, as well as the institutional research ethics boards of each participating site (see the Acknowledgements section for complete list). All involved subjects gave informed consent to participate in the study.

## Results

We identified 1,070 women with SSc, contributing a total of 3,546 observations over the study period, and resulting in a mean number of visits of 3.3 (SD 3.6). Of these women, at baseline, 65% had limited disease and 35% diffuse disease (Table 1). At cohort entry, mean age and mean disease duration were respectively 55.5 (SD 11.7) and 11.2 (SD 9.6) years, and 72% of subjects had already reached menopause. Overall, at baseline, the mean mRSS was 9.6 (SD 9.1), and in pre- and postmenopausal women, it was respectively 12.0 (SD 10.5) and 8.7 (SD 8.4).

**Table 1:** Patients’ characteristics at baseline (n = 1,070)

**Characteristic**	**Value**
Menopausal status, n (%)	
Premenopausal	291 (28.1)
Postmenopausal	744 (71.9)
Disease subtype, n (%)	
Limited	675 (65.0)
Diffuse	369 (35.0)
Age, mean (SD)	55.5 (11.7)
Disease duration, mean (SD)	11.2 (9.6)
Modified Rodnan skin score, mean (SD)	
Overall	9.6 (9.1)
Limited	5.3 (4.2)
Diffuse	17.5 (10.1)
Race/ethnicity, n (%)	
White	900 (84.3)
Black	7 (0.7)
Other	160 (15.0)
Ever smokers, n (%)	576 (53.8)
Medication exposure, n (%)	
Oral contraceptives, current use	21 (2.0)
Hormone replacement therapy, current use	86 (8.2)
DMARDs, current use	183 (17.4)
Cyclophosphamide, ever exposed	85 (8.0)

DMARDs, disease-modifying antirheumatic drugs.

In multivariate analysis, adjusting notably for age, follow-up time, and disease duration, we demonstrated a substantial effect of postmenopausal status on the mean mRSS in women with diffuse disease subtype (−2.62 units, 95% CI −4.44, −0.80; that is, postmenopausal status being associated with a lower mean mRSS compared to premenopausal status in women with diffuse disease), and substantial interaction between menopausal status and disease subtype (2.04 units, 95% CI 0.20, 3.88) (Table 2). In the presence of interaction between two covariates (for example, menopausal status and disease subtype), there is no longer any unique effect of one interacting variable (for example, menopausal status), because its effect depends upon the value of the other interacting variable (for example, disease subtype). Thus, as reflected by the multivariate regression coefficients, and in comparison to the effect seen in women with diffuse SSc, the effect of postmenopausal status on the mean mRSS (compared to premenopausal status) was smaller in women with limited SSc (−0.58, 95% CI −1.50, 0.34) and statistically not significant.

**Table 2:** Univariate and multivariate analyses of covariate effects on the modified Rodnan skin score (n = 1,070)

**Covariate**	**Univariate effect estimate (95% CI)**	**Multivariate effect estimate (95% CI)**
Menopausal status		
Premenopausal	Reference	Reference
Postmenopausal	−2.03 (−3.07, −0.99)	−2.62 (−4.44, −0.80)
Disease subtype		
Diffuse	Reference	Reference
Limited	−10.45 (−11.21, −9.69)	−12.10 (−13.81, −10.39)
Menopause* disease type^a^	1.90 (0.06, 3.74)	2.04 (0.20, 3.88)
Time since baseline (years)	−0.13 (−0.32, 0.06)	0.19 (0.04, 0.34)
Disease duration at baseline (years)	−0.14 (−0.18, −0.10)	−0.05 (−0.07, −0.03)
Age at disease onset (years)	0.04 (0.01, 0.07)	0.02 (−0.01, 0.05)
Race/ethnicity		
White	Reference	Reference
Black	−2.00 (−9.96, 5.96)	−4.6 (−10.11, 0.91)
Other	0.66 (−0.73, 2.05)	−0.46 (−1.42, 0.50)
Smoking		
Never	Reference	Reference
Ever	−0.12 (−1.40, 1.16)	−0.08 (−0.71, 0.55)
Oral contraceptive pill		
Past/never	Reference	Reference
Current	−0.93 (−4.28, 2.42)	−0.60 (−3.03, 1.83)
Hormone replacement therapy		
Past/never	Reference	Reference
Current	−0.99 (−3.05, 1.07)	−0.43 (−1.74, 0.88)
DMARDs^b^		
Past/never	Reference	Reference
Current	2.09 (0.94, 3.24)	1.08 (0.22, 1.94)
Cyclophosphamide		
Never	Reference	Reference
Ever	1.56 (−0.23, 3.35)	0.73 (−0.60, 2.06)

^a^Interaction term between menopausal status and disease subtype. DMARDs, ^b^disease- modifying antirheumatic drugs.

As expected, we observed an important effect of the diffuse disease subtype on the mRSS compared to the limited disease subtype, the former being associated with a higher mean mRSS by 12.10 (95% CI 10.39, 13.81) and 10.06 (95% CI 9.27, 10.85) units in premenopausal and postmenopausal women, respectively. In addition, we found a small effect of time since baseline (0.19 unit, 95% CI 0.04, 0.34) and disease duration at baseline (−0.05 unit, 95% CI −0.07, −0.03), and a moderate effect of DMARDs (1.08 unit, 95% CI 0.22, 1.94) on the mean mRSS.

We did not observe an effect of smoking on the mean mRSS (−0.08 unit, 95% CI −0.71, 0.55) and any significant confounding of smoking on the effect of menopause (when comparing the univariate and multivariate effect estimates and CI). Moreover, sensitivity analyses using different smoking exposure definitions (that is, defining exposure alternatively as 1) current, past, or never smoker, and 2) current or past/never smoker) did not reveal any further confounding of smoking on the main effect estimate (data not shown).

In the multivariate analysis restricted to women with early disease (that is, baseline disease duration shorter than 5 years) (Table 3), we observed a larger effect of postmenopausal status on the mean mRSS (compared to premenopausal status) both in women with diffuse (−3.36 units, 95% CI −5.87, −0.85) and limited SSc (−1.45, 95% CI −3.21, 0.31), as opposed to the effect estimates observed in the overall sample.

**Table 3:** Multivariate analysis of covariate effects on the modified Rodnan skin score in a subsample of women with early disease (n = 370)

**Covariate**	**Multivariate effect estimate (95% CI)**
Menopausal status	
Premenopausal	Reference
Postmenopausal	−3.36 (−5.87, −0.85)
Disease subtype	
Diffuse	Reference
Limited	−13.50 (−16.10, −10.99)
Menopause*disease type^b^	1.91 (−0.83, 4.65)
Time since baseline (years)	−0.13 (−0.40, 0.14)
Disease duration at baseline (years)	−0.47 (−0.98, 0.04)
Age at disease onset (years)	0.04 (0.00, 0.08)
Race/ethnicity	
White	Reference
Black	−0.64 (−2.85, 1.11)
Other	−0.87 (−1.42, 0.50)
Smoking	
Never	Reference
Ever	0.86 (−0.55, 2.15)
Oral contraceptive pill	
Past/never	Reference
Current	−0.78 (−2.15, 0.43)
Hormone replacement therapy	
Past/never	Reference
Current	0.08 (−2.84, 3.00)
DMARDs^c^	
Past/never	Reference
Current	0.10 (−1.61, 1.81)
Cyclophosphamide	
Never	Reference
Ever	−0.33 (−2.31, 1.65)

^b^Interaction term between menopausal status and disease subtype. DMARDs, ^c^disease- modifying antirheumatic drugs.

## Discussion

We found that postmenopausal status in women with diffuse SSc was associated with a substantially lower mean mRSS (by −2.62 units, 95% CI −4.44, −0.80) compared to premenopausal status. This effect was independent of age, follow-up time, and disease duration. Our findings are supported by previous experimental evidence showing that estrogen increases extracellular matrix protein production in skin fibroblast culture of SSc patients [9]. In addition, observational studies of healthy women showed skin thinning in postmenopausal women compared to women who had not yet reached their menopause [10]. Furthermore, it is well-established that estrogen stimulates normal skin fibroblasts to produce transforming growth factor-beta 1 (TGF-beta 1), as well as monocytes and macrophages to produce platelet-derived growth factor (PDGF). Both TGF-beta 1 and PDGF are key profibrotic cytokines in SSc skin disease [1,16]. As SSc skin fibroblasts show increased expression of TGF-beta 1 receptor and PDGF receptor, estrogen might play a role in SSc pathogenesis through its stimulatory effect on these two cytokines.

The effect of postmenopausal status on skin thickening was smaller in women with limited SSc compared to women with diffuse SSc, as evidenced by the interaction term between menopausal status and disease subtype. One potential explanation for the differential effect of menopause on skin thickening in women with diffuse and limited SSc might reside in the disease subtypes definition. Indeed, disease subtypes are defined according to the extent and location of skin involvement (for example, in the limited subtype skin is affected in the hands, feet, forearms, and/or face) [17]. Thus, by definition, the mRSS is usually lower in subjects with limited SSc than in those with diffuse SSc, with different maximum scores (that is, respectively 27 and 51 units). This might explain at least in part the smaller (absolute) effect size of menopausal status. Indeed, when contrasted to the baseline mean mRSS, the (relative) effect size of menopausal status was comparable between diffuse and limited subtypes, representing respectively 15% and 10% of the subtype-specific mean baseline mRSS.

As expected and shown in prior studies [2], we observed that disease subtype was the strongest predictor of skin thickening in our cohort, with diffuse SSc being associated with a higher mean mRSS of at least 10 units. We also found more severe skin thickening in women exposed to DMARDs. However, this is likely to have reflected confounding by disease severity (also known as confounding by indication), which occurs when a medication is preferentially prescribed to a group of patients with a worse baseline prognosis [18]. Indeed, SSc subjects with rapidly progressing and/or more extensive skin thickening are more likely to have higher mRSS and be aggressively treated with DMARDs, potentially resulting in a seemingly positive effect of DMARD exposure on the mRSS.

Although not statistically significant, women exposed to OCP and HRT appeared to have less skin thickening than unexposed women. Based on available evidence showing that exogenous estrogen increases skin thickness in normal women, we would have expected the opposite [10,12]. Still, this finding could also represent confounding by disease severity, in which case, women with less severe skin disease and fewer disease complications (such as pulmonary hypertension or cardiovascular disease) would be more likely to be sexually active (and be on OCP) or have fewer contraindications to OCP and/or HRT.

The magnitude of the effect of postmenopausal status on the mRSS in women with diffuse SSc is very interesting, considering that menopause occurs in all women and SSc predominantly affects women. Very few exposures, including medications, seem to alter the course of skin thickening in SSc. The minimally important difference in the mRSS has been established at 3.2 units, based on a prior randomized controlled trial of d-penicillamine in subjects with diffuse disease [19]. As the CSRG cohort is not an inception cohort, women included in our study had long disease duration. Since it is well-established that skin thickening progresses more rapidly in early than in late disease [1,2], inclusion of women with longer disease duration might have limited our ability to observe a larger effect of menopausal status on skin thickening. This was evidenced by our subsample analysis of women with disease duration shorter than 5 years, showing a larger effect of postmenopausal status on the mRSS in both disease subtypes. Notably, the magnitude of the postmenopausal effect in diffuse SSc (−3.36 units, 95% CI −5.87, −0.85) exceeded the minimally important difference in the mRSS [19].

Medsger *et al*. described the natural evolution of skin thickening in SSc subjects [20]. After rapid skin thickening progression in the first 5 years of the disease, in limited SSc, skin thickening slows for a few years to reach a plateau after 10 years of evolution, whereas in diffuse SSc, skin thickening actually regresses, albeit not to the pre-disease state [20]. Interestingly, in our study, the effect estimates for the covariates, time since cohort entry (that is, follow-up time) and disease duration at baseline, reflected this evolution/involution. Indeed, time since cohort entry had an overall positive effect on skin thickening (that is, each additional year of follow up being associated with an average increase of 0.19 unit (95% CI 0.04, 0.34)), because with time skin thickening increases more than it regresses. However, disease duration at baseline had an overall negative effect on the mRSS (that is, each additional year of disease duration at baseline being associated with a lower mean mRSS by −0.05 unit (95% CI −0.07, −0.03)), as skin thickness peaks in women with early disease. Moreover, the negative effect of menopausal status on the mRSS that was objectivated in our study might explain, at least in part, the natural plateau and/or involution of skin thickening reported by Medsger *et al*., which could have coincided with menopause onset in some subjects [20]. In addition, we did not find an association between age at disease onset and skin thickening after accounting for menopausal status in multivariate analysis, suggesting that menopausal status might be a better predictor of skin thickening compared to age at diagnosis.

Our study has some potential limitations. We used the total mRSS as the outcome measure, which assesses both the extent and degree of skin thickening. Thus, we could not distinguish the association between menopausal status and the extent of skin thickening from the association between menopausal status and the degree of skin thickening. In addition, we were unable to definitively demonstrate an effect of smoking on skin thickening. Previous work from our group has shown the challenges of appropriately modeling smoking exposure in health outcome analyses of smoking effect in SSc patients, and how these analyses can suffer from the healthy smoker effect and/or causality bias, making the smoking effect-estimate more conservative than it should be (that is, biasing the effect estimate towards the null) [21]. Still, this is unlikely to have affected our main effect-estimate as smoking did not appear to be a strong confounder of the effect of menopausal status on skin thickening as shown in the univariate and multivariate analyses. Another potential limitation is that menopausal status was ascertained annually by self-report and not confirmed by any laboratory investigation. However, a previous study has shown high validity of self-reported menopausal status and suggested self-report as a sufficiently accurate measure of menopause in observational studies [22].

## Conclusions

Our results suggest that menopause has a substantial effect on skin thickening in diffuse SSc, with postmenopausal status being associated with a lower mean mRSS compared to premenopausal status. This effect might be more pronounced in early disease (that is, baseline disease duration shorter than 5 years). Our findings should prompt further research on the role of estrogen on skin disease progression in SSc.

## Abbreviations

CSRG: Canadian Scleroderma Research Group; DMARDs: disease-modifying antirheumatic drugs; GEE: generalized estimating equation; HRT: hormone replacement therapy; mRSS: modified Rodnan skin score; OCP: oral contraceptive pills; PDGF: platelet-derived growth factor; SSc: systemic sclerosis; TGF-beta 1: transforming growth factor-beta 1.

## Competing interests

All authors declare that they have no competing interests.

## Authors’ contributions

EV conceived of the study and participated in its design, performed the statistical analyses, and drafted the manuscript. SB participated in the study design, data analysis, and helped draft the manuscript. MH participated in the study design, data analysis, and helped draft the manuscript. CAP participated in the study design, data analysis, and helped draft the manuscript. MB participated in the study design, data analysis, and helped draft the manuscript. All investigators of the CSRG group contributed to data collection and critically revised the manuscript. All authors approved the final version and take full responsibility for the manuscript.
